# Thoughts of suicide or self-harm among healthcare workers during the COVID-19 pandemic: qualitative analysis of open-ended survey responses

**DOI:** 10.1192/bjo.2022.509

**Published:** 2022-06-14

**Authors:** Marie Bismark, Natasha Smallwood, Ria Jain, Karen Willis

**Affiliations:** Centre for Health Policy, The University of Melbourne, Australia; Department of Respiratory Medicine, The Alfred Hospital, Australia; and Department of Allergy, Immunology and Respiratory Medicine, Central Clinical School, The Alfred Hospital and Monash University, Australia; Faculty of Medicine, Nursing and Health Sciences, Monash University Clayton, Australia; Institute for Health and Sport, Victoria University, Australia

**Keywords:** COVID-19, suicide, self-harm, healthcare workers, patient safety

## Abstract

**Background:**

Healthcare workers are at higher risk of suicide than other occupations, and suicidal thoughts appear to have increased during the COVID-19 pandemic.

**Aims:**

To understand the experiences of healthcare workers with frequent thoughts of suicide or self-harm during the pandemic, including factors that contributed to their distress, and the supports that they found helpful.

**Method:**

We used content analysis to analyse free-text responses to the Australian COVID-19 Frontline Healthcare Workers Study, from healthcare workers who reported frequent thoughts that they would be better off dead or of hurting themselves, on the Patient Health Questionnaire-9.

**Results:**

A total of 262 out of 7795 healthcare workers (3.4%) reported frequent thoughts of suicide or self-harm in the preceding 2 weeks. They described how the pandemic exacerbated pre-existing challenges in their lives, such as living with a mental illness, working in an unsupportive environment and facing personal stressors like relationship violence or unwell family members. Further deterioration in their mental health was triggered by heavier obligations at home and work, amid painful feelings of loneliness. They reported that workplace demands rose without additional resources, social and emotional isolation increased and many healthful activities became inaccessible. Tokenistic offers of support fell flat in the face of multiple barriers to taking leave or accessing professional help. Validation of distress, improved access to healthcare and a stronger sense of belonging were identified as helpful supports.

**Conclusions:**

These findings highlight the need for better recognition of predisposing, precipitating, perpetuating and protective factors for thoughts of suicide and self-harm among healthcare workers.

## Impact of crisis events on healthcare workers

During the COVID-19 pandemic, rates of suicidal and self-harm ideation appear to have increased among healthcare workers. For example, a study of healthcare workers from nine intensive care units in the UK found that 13% had contemplated suicide or self-harm during the pandemic,^1^ with rates of suicidal ideation among healthcare workers estimated at between 3.6 and 11.1% in studies from Europe and Asia.^2–5^ In Australia, the Frontline Healthcare Workers Study – an online survey of nearly 10 000 healthcare workers across Australia – found that around one in ten reported thoughts of suicide or self-harm over a 2-week period during the second wave of the pandemic.^6^

Although these figures are deeply concerning, they align with prior understandings of clinician health and the impact of crisis events on front-line workers. Before the pandemic, healthcare workers were known to have elevated rates of depression, anxiety, traumatic stress conditions and thoughts of suicide and self-harm, compared with the general population.^7^ In Australia, the Beyond Blue study found that 10.4% of doctors experienced thoughts of taking their own life over a 12-month period.^8^ A large national study of coronial data^9^ found that female healthcare professionals, and male nurses and midwives, have higher rates of death by suicide than workers in other occupations. These high rates of psychological distress have been attributed to occupational factors, including high work demands, poor organisational support, long hours and exposure to workplace violence.^10,11^ Among healthcare workers who die by suicide, access to lethal means of suicide by way of prescription medicines also plays a role.^9^

Crisis events are also associated with a deterioration of mental health among front-line workers.^12^ Pre-existing stressors, such as long working hours, often worsen during a crisis and can be compounded by the moral injury of having to act in ways that transgress deeply held values.^13^ Work-related mental health impairment was identified among healthcare workers after the 2003 severe acute respiratory syndrome pandemic,^12^ Hurricane Katrina^14^ and the Fukushima nuclear accident in Japan.^15^ During the current pandemic, healthcare workers in Australia^16^ and internationally^17^ have reported high levels of depression, anxiety, burnout and post-traumatic stress disorder symptoms.

## Understanding healthcare workers with thoughts of suicide or self-harm

A deeper understanding of the experiences of healthcare workers with thoughts of suicide or self-harm would help to identify healthcare workers at risk, and inform interventions to support their health and well-being. However, they are a hard to reach group: many healthcare workers are reluctant to disclose thoughts of suicide or self-harm because of ongoing stigma around mental illness, misplaced fears of mandatory reporting to their professional regulator^18^ and concerns about their professional reputations.^19^

This study explores their experiences of living and working through the pandemic, the factors that contributed to their distress and the supports that they identified as helpful.

## Method

### Study design

We analysed free-text responses from the Australian COVID-19 Frontline Healthcare Workers Study, to explore the perspectives and experiences of healthcare workers with frequent thoughts of suicide or self-harm during the pandemic. Although quantitative research is useful for calculating rates and estimating risks for thoughts of suicide and self-harm, qualitative research enables more complex and contextualised understandings of subjective experiences.^20^

Detailed information about the survey method, along with findings from quantitative analyses, is published elsewhere.^16^ In summary, the study was an Australia-wide, voluntary, anonymous online survey of healthcare workers from all professional backgrounds and roles, between August and October 2020. Participants did not need have cared for patients with COVID-19 to participate and no incentives were offered to participate.

Data collected included quantitative measures of participant demographics, living and workplace situations, and psychometric scales to assess mental health symptoms. The survey also included four free-text questions (see Box 1). These questions provided an opportunity for healthcare workers to share their experiences of work and life during the pandemic.

Box 1 Free-text questions included in the Frontline Healthcare Workers Study

1. What do you think would help you most in dealing with stress, anxieties and other mental health issues (including burnout) related to the COVID-19 pandemic?

2. What did you find to be the main challenges that you faced during the COVID-19 pandemic?

3. What strategies might be helpful to assist front-line healthcare workers during future crisis events like pandemics, disasters etc?

4. Is there anything else that you would like to tell us about the impact of the COVID-19 pandemic or regarding supports that you feel are useful for well-being?

### Identification of participants

The sub-analysis reported in this paper focuses on those survey respondents who answered the Patient Health Questionnaire-9 (PHQ-9) question about ‘thoughts that [they] would be better off dead or of hurting [themselves] in some way’ with a response of ‘nearly every day’ (*n* = 99) or ‘more than half the days’ (*n* = 163). Analysis of this subgroup allowed us to capture insights from a hard-to-reach population of healthcare workers with frequent thoughts of suicide or self-harm, working in diverse roles (e.g. nurses, doctors, allied health practitioners and administrative staff) and settings (e.g. public and private, hospital-based and community care, metropolitan and rural) across Australia.

### Analysis

Demographic characteristics of our sample are reported descriptively, alongside the characteristics of healthcare workers who did not experience frequent thoughts of suicide or self-harm. We used chi-squared tests to calculate *P-*values, with significance indicated by *P* ≤ 0.05. Data analysis was performed with Stata statistical software (StataCorp, release 17 for Windows). More detailed quantitative analyses from the Frontline Healthcare Workers Study, including multivariable predictors of thoughts of suicide or self-harm, are reported elsewhere.^6^

Our qualitative descriptive study used content analysis^21^ to analyse participants’ responses to the four free-text questions. Content analysis provides a strategy for capturing the meanings within data, including data from questionnaires,^20^ and is useful when little is known about a particular phenomenon in a selected group of people.

The research team included three authors with a clinical background: M.B. is an advance trainee in psychiatry, R.J. is a medical student and N.S. is a consultant respiratory physician. The fourth author, K.W., is a health sociologist and qualitative research methodologist.

Responses from all 262 healthcare workers with frequent thoughts of suicide or self-harm were imported into Microsoft Excel (Excel 2021 for Windows) for coding. All responses were independently coded by two researchers (M.B. and R.J.). The coding taxonomy was developed inductively. Following initial coding, the data within each code were sorted and compared, identifying patterns and differences in ideas and concepts.^21^ Codes were sorted into categories or key ideas at a manifest level. Finally, the categories were reflected on, and grouped into four themes, based on threads running through the key ideas on an interpretive level.^22^ To enhance trustworthiness, codes, key ideas, and themes were discussed during regular meetings with co-authors (K.W. and N.S.). Any differences in interpretation were resolved by consensus.

We found a strong commonality of key ideas across participants from all roles and areas of work across the health sector. In developing our themes, we drew on a widely used approach to psychiatric formulation that asks, ‘Why is this person unwell, in this way, at this time?’, within a framework of ‘4Ps’:^23^ predisposing factors put someone at risk of developing a problem, precipitating factors trigger the onset of a problem, perpetuating factors maintain the problem once it has been established and protective factors reduce the severity of the problem. The incorporation of a formulation approach in developing our themes emerged in response to initial coding of the data, rather than being an *a priori* decision, and was informed by M.B.'s training and experience in psychiatry.

Quotes from participants are used to illuminate key themes, with some demographic information also provided.

### Ethics

The authors assert that all procedures contributing to this work comply with the ethical standards of the relevant national and institutional committees on human experimentation and with the Helsinki Declaration of 1975, as revised in 2008. Written consent was obtained from all participants. All procedures involving human participants were approved by the Royal Melbourne Hospital Human Research Ethics Committee (approval number HREC/67074/MH-2020).

## Results

Overall, 7795 healthcare workers provided complete survey responses including the non-mandatory question on thoughts of suicide or self-harm. Among these, 819 reported thoughts of suicide or self-harm in the preceding two weeks. For 557 participants these thoughts were ‘occasional’; 262 participants reported ‘frequent’ thoughts of suicide or self-harm, of whom 212 provided free-text responses. These free-text responses included forthright descriptions of ‘severe depression and suicidality’ and the ‘abysmal state’ of their mental health during the pandemic, aligning with their responses to the PHQ-9.

‘I spoke to my 24-year-old son about my death … I'm no longer compassionate which has never been me. I really just want to stay home, I'm tired, lonely & sad and even writing this makes me feel guilty.’ Nurse, community care, female, age 51–64 years.

‘[I have] overwhelming and persistent feelings of pointlessness and hopelessness.’ Senior doctor, anaesthetics, male, age 31–40 years.

Participants reporting frequent thoughts of suicide or self-harm, were more often male, in a younger age group, living alone and had a previous history of mental illness (Table 1).

**Table 1:** Characteristics of healthcare workers with and without frequent suicidal ideation

Characteristic		Nil or occasional thoughts of suicide or self-harm (*N* = 7533)	Frequent thoughts of suicide or self-harm (*N* = 262)	*P*-value
		*n* (%)	*n* (%)	
Gender	Male	1386 (18%)	66 (25%)	<0.0001
	Female	6109 (81%)	191 (73%)	
	Non-binary/prefer not to say	38 (<1%)	5 (2%)	
Age	≤30 years	1759 (23%)	89 (34%)	<0.0001
	31–40 years	2155 (29%)	82 (31%)	
	41–50 years	1691 (22%)	39 (15%)	
	>50 years	1928 (26%)	52 (20%)	
Profession	Medicine	2379 (32%)	68 (26%)	0.070
	Nursing	2938 (39%)	115 (44%)	
	Allied health	1517 (20%)	47 (18%)	
	Other	698 (9%)	32 (12%)	
Living situation	Living alone	1026 (14%)	52 (20%)	0.006
	Living with others	6507 (86%)	210 (80%)	
Previous mental illness	Yes	2208 (29%)	167 (64%)	<0.001
	No	5325 (71%)	95 (36%)	
Increased income worries	Yes	2260 (30%)	137 (52%)	<0.001
	No	5273 (70%)	125 (48%)	

### Themes

**Fig. 1:**
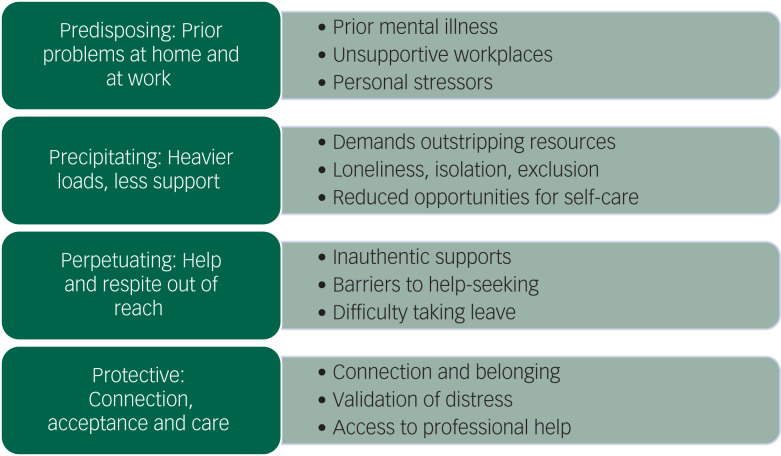
Themes reported by healthcare workers with frequent thoughts of suicide or self-harm.

Corresponding with the 4Ps framework, we identified four themes (Fig. 1). First, healthcare workers with thoughts of suicide or self-harm spoke about already being vulnerable owing to prior problems at work and at home. Second, the deterioration in their mental health was often triggered by heavier workloads and less support during the pandemic. Third, recovery from thoughts of suicide or self-harm was impeded when opportunities to rest and access help were out of reach. Finally, healthcare workers spoke about the healing power of connection, acceptance and care. Key ideas were nested within each of these four themes, as described below.

### Prior problems at home and at work

Healthcare workers with thoughts of suicide or self-harm spoke about the interrelationship between personal difficulties, which often predated the pandemic, and the stresses of the pandemic.

#### History of mental illness

In their survey responses, two-thirds of participants disclosed a history of mental illness and several referred to this history in their free-text responses.

‘I have previously been diagnosed with generalised anxiety disorder, but pre-COVID I was able to manage with healthy coping strategies and psychology. Since COVID I have been diagnosed with depression and worsening anxiety and commenced medication.’ Nurse, intensive care, female, age 31–40 years.

‘The depression I was diagnosed with before the pandemic hit, has unfortunately completely spiralled out of control to the point of suicidality.’ Junior doctor, emergency department, female, age 31–40 years.

#### Personal stressors

Participants wrote about significant stressors in their personal lives, which may have occurred with or without the pandemic, but were exacerbated by the pandemic. These included domestic violence and other relationship problems, illness or death within the family, the stress of tertiary study and financial insecurity.

‘I think the hardest part is actually coping with all the other non-pandemic issues that would have occurred regardless. Things like my father's ill health, my husband having a heart condition diagnosed, building a house … Adding all those things onto a challenging work environment, curfews, constant anxiety … I think the challenges of COVID-19 make everything else that has been brewing surface and blow out of proportion.’ Nurse, hospital-based, female, age 20–30 years.

‘Difficult teenage behaviours with son using cannabis and engaging in problematic behaviours … leading to arguments and distance with husband. … Difficulties I have encountered have been within the home and exacerbated by work.’ Nurse, hospital-based, female, age 51–64 years.

#### Unsupportive workplace

When describing the challenges of the pandemic (question 2), many healthcare workers wrote about feeling undervalued and unsupported at work.

‘I'm now in a state of “learned helplessness” … every time we tried to change or do the right thing to protect our staff it was a fight to get the appropriate changes.’ Senior doctor, anaesthetics, female, age 51–64 years.

‘I feel like my workplace doesn't care about its employees and will work us until we burn out or kill ourselves.’ Nurse, intensive care, female, 20–30 years.

Although the wider cohort of survey respondents commonly described feelings of ‘camaraderie’, ‘collegiality’ and ‘team support’ in their workplace,^24^ such descriptions were strikingly absent among the healthcare workers with frequent thoughts of suicide or self-harm. Indeed, only three described positive experiences at work during the pandemic in their free-text responses.

### Heavier load and less support

Participants wrote about rapid changes within the health sector and wider society leading to heavier burdens at work and at home. These burdens were rarely matched with additional support or resources. Additionally, many of their previous supports, including social connections and self-care strategies, were stripped away by public health restrictions.

#### Demands outstripping resources

A third of the participants wrote about greater demands at work, with many commenting on the challenges of meeting this ‘increased workload with inadequate staff’ (Nurse, emergency department, female, age 20–30 years). Workplace pressures, patient acuity and the pace of change all increased during the pandemic, and for most healthcare workers there was no commensurate increase in resources, training or pay.

‘The combination of increased workload, increased responsibility, and decreased support and supervision has been very challenging.’ Junior doctor, medical specialty, female, age 20–30 years.

‘I work night shift [in the emergency department and] the bulk of the presentations have been related to mental health crises - yet there remains no increase in the number of mental health triage nurses overnight. One person to manage up to 15 presentations overnight is not fair or safe to manage on their own.’ Nurse, emergency department, female, age 20–30 years.

These increased demands sometimes had devastating flow-on effects in other aspects of healthcare workers’ lives.

‘[A] physically extremely demanding job, not able to take breaks, working many, many unpaid overtime hours - leading to pure exhaustion … My workplace forced me to stop breastfeeding - I was told that they couldn't afford for me to have a pumping break [and] thus had to stop breastfeeding my son prematurely. I was not at all ready for him to be weaned … I have suffered a severe depressive episode [and] due to the new medication I am on, I have had to indefinitely cease trying to conceive baby number two.’ Nurse, surgical, female, age 31–40 years.

Other healthcare workers spoke about their feelings of moral distress when the demands on the healthcare system outstripped what they could, or were permitted to, provide.

‘I could no longer help people which is what I do for a job. So many restrictions and doctors/services refusing to see patients. I am deeply concerned that patients are delaying or not receiving appropriate care which is making for poorer outcomes.’ Nurse, community care, female, age 31–40 years.

‘Watching COVID patients die without family present. Watching non-COVID patients acutely unwell in the intensive care unit not being able to have visitors unless they were being palliated.’ Nurse, intensive care, female, age 20–30 years.

Increased job demands without a commensurate increase in resources led some participants to consider leaving their profession.

‘We're all just exhausted. I was burnt out pre-pandemic and now feel so guilty about wanting to leave medicine. I will stay because I feel I need to, not because it's what's best for me.’ Junior doctor, emergency department, female, 31–40 years.

‘This has destroyed bedside nursing for me, and for the sake of my mental health I will have to leave the field once I mentally accept that I can longer help others, because no one has helped us when we needed it.’ Nurse, aged care, female, 20–30 years.

#### Feeling alone

One of the strongest themes in participants’ responses was the profound sense of aloneness that many described. Over a quarter of healthcare workers with frequent thoughts of suicide or self-harm wrote about feelings of loneliness, separation, disconnection or exclusion.

‘Social isolation. I haven't seen a smile on a person's face not through a computer screen in months.’ Nurse, intensive care, female, age 31–40 years.

These feelings of aloneness were multifaceted, and arose from multiple sources, including personal living situations, decisions to self-isolate from others, travel restrictions and border closures, loss of camaraderie at work, increased bullying and exclusion in workplaces, and feeling ostracised by members of the public (Table 2).

**Table 2:** Causes of healthcare workers feeling alone during the pandemic

Cause of feeling alone	Description	Illustrative quotes
Lonely	Living alone and unable to see others because of lockdown rules	‘I am single and do not have housemates. All the apps, support lines and face timing cannot counteract the complete loneliness experienced. For me the only thing that would have made a slight difference would have been social contact.’ Junior doctor, emergency department, female, age 31–40 years
Separated	Unable to visit loved ones because of travel restrictions and border closures	‘[I'm on] a temporary working visa and unable to see my 5-year-old son who is stuck in another country.’ Student, general medicine, female, age 31–40 years
Secluded	Voluntarily living away from family to reduce the risk of infecting others	‘The anxiety and isolation that comes with being a high-risk spreader for COVID-19. Have been too worried about giving it to my loved ones that I haven't seen my partner in 2 months or any of my friends.’ Nurse, medical specialty, female, age 20–30 years
Unsupported	Feeling that no-one cares and no-one is helping them	‘No-one is actually there to support. Everyone seems to offer support, until you ask for it. Then they offer nothing.’ Junior doctor, emergency department, male, age 31–40 years
Disconnected	Missing workplace camaraderie because of closure of tearooms, personal protective equipment and social distancing	‘The biggest thing was taken away, the ability to be social and debrief after a shift together. This has largely been dehumanising. The social acceptance and togetherness have been stripped away.’ Technician, emergency department, male, age 41–50 years
Excluded	Experiencing an increase in workplace bullying and exclusion	‘Lack of help from colleagues, bullying, harassment. Exclusion behaviour existing prior to COVID-19 increased.’ Clinical scientist, medical speciality, female, age 51–64 years
Ostracised	Feeling stigmatised by members of the public	‘The public will thank healthcare workers but then move away from you on a tram, or you get dirty looks when having a break in uniform. Very isolating.’ Nurse, medical speciality, female, age 20–30 years

#### Reduced opportunities for self-care

Participants wrote about the difficulty of managing their distress when usual practices of self-care were profoundly disrupted by public health restrictions. Many of their usual ways of ‘nurturing self’, ranging from indoor exercise to social gatherings with friends, could no longer be accessed resulting in a perceived ‘inability to do things that help de-stress’.

‘Severe relapse of mental health conditions and unable to access usual supports (seeing friends/family, engaging in relaxing activities outside the home).’ Speech pathologist, hospital aged care, female, age 31–40 years.

‘My best coping mechanism was training in martial arts; that has been taken away. If you continually strip coping mechanisms from people, are you really surprised that people are struggling to deal with the world?’ Technician, emergency department, male, age 41–50 years.

### Help and respite out of reach

#### Barriers to help-seeking

When asked about the biggest challenges posed by the pandemic (question 2), participants identified many barriers to seeking help, including limited time, cost, stigma and insincere offers of help.

Participants explained how time constraints prevented help-seeking, because of an increased workload and staffing shortages during the pandemic.

‘I think I should talk to a professional but part of me feels scared or feels I have no time to do so. And I feel I cannot take a mental health day as we are short on staff; that'd make me feel irresponsible if I took a day off.’ Junior doctor, emergency department, female, age 20–30 years.

Several participants wrote about financial barriers to accessing psychological supports.

‘More counselling [would help]. I got only six [sessions] from my private health insurance.’ General practitioner, COVID screening, male, age 51–64 years.

‘The hospital should put in place more free mental health support services for healthcare workers because we do experience a lot, and it would be nice to be able to talk to someone professional to debrief.’ Junior doctor, emergency department, female, age 20–30 years.

For some, the stigma of mental illness, particularly in healthcare workplaces, was a barrier to seeking mental health support.

‘Nurses are afraid to disclose if they are having difficulty coping or are anxious/depressed.’ Nurse, community care, female, age 31–40 years.

‘Pre the pandemic you would risk being bullied out of the health service if you disclosed any mental health issue/chronic health condition/injury. This risk [has] exponentially increased since the pandemic.’ Physiotherapist, female, age 41–50 years.

#### Inauthentic or inappropriate supports

Many participants felt that workplace psychological supports implemented during the pandemic missed the mark in meeting their needs. Some healthcare workers perceived that offers of help were ‘tokenistic and disingenuous’ – designed to make their employer ‘look good or appear supportive, but actually they aren't’. As a result, they felt abandoned and alone.

‘Within the environment I work, it is more common to kick someone when they are down rather than help.’ Nurse, emergency department, female, age 51–64 years.

‘For me all the apps, emails, and support unfortunately have felt hollow. In the end you have to do it alone.’ Junior doctor, emergency department, female, age 31–40 years.

#### Difficulty taking leave

One in five participants wrote about the importance of taking ‘time out’ and having access to ‘paid leave for the specific purpose of rest and recuperation’ to manage their psychological distress.

‘So many people have needed to take a day or two for their own mental health but have not been able to.’ Nurse, emergency department, female, age 20–30 years.

They wrote about being pushed to keep working, after running out of leave or having leave requests denied.

‘Severe burnout which I expressed to my manager [was] not recognised. [I was] ignored and given zero support or time off despite repeated requests; ended up having a breakdown and needing to use personal leave/stress leave.’ Nurse, respiratory medicine, female, age 31–40 years.

‘[We need] jobs with leave entitlements so that healthcare workers can take leave when they are unwell rather than going to work sick or risk losing their income.’ Junior doctor, medical speciality, female, age 31–40 years.

For some, the greatest barrier to taking leave was concern about shifting the burden onto those colleagues who remained.

‘I feel I cannot take a mental health day as we are short on staff; that'd make me feel irresponsible if I took a day off.’ Junior doctor, emergency department, female, age 20–30 years.

For others, who were on casual contracts or had used up their paid leave, financial stressors were a major barrier to taking leave, despite their own poor health and the needs of their families. As shown in Table 1, over half of the healthcare workers with frequent thoughts of suicide or self-harm reported increased income worries during the pandemic.

‘More financial … support [would help]. If it was possible to have a week's paid leave to spend some time with my family and not having to worry about work. Or maybe an extra week's pay/bonus to help with the cost of rising bills. My 12-year-old son some days is schooling from home by himself. If I don't go to work, I don't get paid.’ Administrative worker, community care, female, age 31–40 years.

### Connection, acceptance and care

The final theme captured healthcare workers’ views on the importance of connection, acceptance and care as protective factors against psychological distress during the pandemic.

#### Belonging and connection

Many of the healthcare workers with thoughts of suicide or self-harm spoke about their desire for a stronger sense of connection and belonging within the workplace.

‘[I need] a safe space to share my struggles with colleagues. Fostering compassion for all in the workplace.’ Speech pathologist, hospital aged care, female, age 31–40 years.

‘[It would be helpful to have a] ‘buddy’ system where you work closely with a mutually agreed staff member/colleague for mental, emotional, and work support.’ Nurse, respiratory medicine, female, age 20–30 years.

#### Validation of distress

For some healthcare workers with thoughts of suicide or self-harm, feeling accepted and understood was perceived as more helpful than ‘Band-Aid fixes’ that failed to acknowledge the depth of their distress. Some healthcare workers with frequent thoughts of suicide or self-harm valued knowing that others were also struggling as this lessened the stigma of their experience.

‘Knowing that I am not one of the few suffering, but more part of the majority of how healthcare workers are experiencing this, makes me feel less alone and less ‘defective’ as a doctor.’ Senior doctor, community care, female, age 41–50 years.

Others thought it would be helpful to be able to confide in someone without judgement.

‘[It would help to] be able to talk to someone about these [mental health] issues in a non-judgemental situation.’ Senior doctor, general medicine, male, age 41–50 years

‘Someone to listen to my worries without judgement.’ Pharmacist, female, age 31–40 years.

#### Professional care

Fewer than half of the healthcare workers with frequent thoughts of suicide or self-harm had sought professional help from a psychiatrist, psychologist or general practitioner. Help-seeking was more common among those with a history of mental illness, suggesting that their engagement with professional services began before the pandemic. Some participants who had sought professional help spoke highly of the benefit.

‘Without my psychiatrist's support I think I would not have been here, and I have never ever considered that.’ Clinical scientist, community care, female, age 51–64 years.

## Discussion

Even before the pandemic, healthcare workers were at increased risk of thoughts of suicide and self-harm, and had higher rates of completed suicide than workers in other occupations. The pandemic has amplified these risks. Our findings report on a subset of participants in a large national survey of Australian healthcare workers during the COVID-19 pandemic: those who reported that, on most days of the preceding 2 weeks, they had thoughts that they would be better off dead or of hurting themselves.

Overall, 262 out of 7795 healthcare workers (3.4%) reported frequent thoughts of suicide or self-harm in the 2 weeks before the survey. By comparison, a survey of the general Australian population during the first wave of the pandemic found that 5.7%^25^ of people reported frequent thoughts of suicide and self-harm on the same PHQ-9 question. This difference may reflect sampling differences between the two surveys, or the greater impact of stringent lockdowns and job losses on non-healthcare workers during the early months of the pandemic.

Although the circumstances that lead to thoughts of suicide or self-harm among healthcare workers are multifaceted and deeply personal, we identified important recurring themes. Participants with frequent thoughts of suicide or self-harm described how the COVID-19 pandemic compounded prior problems in their lives, such as invalidating work environments, financial insecurity, poor mental health, weighty carer responsibilities or relationship violence. Further deterioration in their mental health was precipitated by having to carry a heavier burden at home and at work, along with profound feelings of loneliness. Commonly mentioned stressors included inadequate staffing and resources to match the spike in work demands, increased social and emotional isolation owing to staff working from home and closure of shared spaces, and an inability to access restorative activities such as going to the gym or spending time with loved ones. Offers of support were often perceived as tokenistic or out of touch, and complex barriers obstructed efforts to take leave or access professional help. Healthcare workers identified potentially helpful supports, including empathic validation of their distress, improved access to mental healthcare and a stronger sense of belonging and connection with peers.

The predisposing factors identified by our research are concordant with prior research among the general population, and among healthcare workers before the pandemic. For example, mental illness,^26^ unsupportive workplaces and personal stressors such as domestic violence^27^ and bereavement^28^ have all been identified as risks for suicidal ideation. Our research suggests that these factors are also important considerations among healthcare workers during the COVID-19 pandemic.

Our research also provides important insights into the acute stressors that may precipitate thoughts of suicide or self-harm, and in particular, the danger of placing demands on an individual that greatly exceed the physical, psychological and social resources available to them at that time. During the pandemic, healthcare workers around the world have described increased workloads,^29^ limited resources and rapidly changing policies and processes.^30^ Importantly, these increased demands were accompanied by a reduction in social connections because of public health restrictions associated with the pandemic. Over 120 years ago, Durkheim^31^ recognised the association between suicide and loss of social integration, in what he called egoistic suicides, and since that time a growing body of empirical research has underscored the connection between loneliness and suicidal ideation.^32^ In addition, healthcare workers were unable to access tried and true methods of self-care.^33^

Our findings in relation to help-seeking are consistent with previous research showing that long work hours, shift work and on-call arrangements can all make it difficult for healthcare workers to attend regular medical appointments.^34^ Although stigma around mental illness is reducing in society, healthcare workers still describe personal and professional stigma, which makes it more difficult to seek help.^35^ These barriers to accessing help existed before the pandemic,^36^ but may have been exacerbated during the pandemic. Our findings echo calls from within the health profession for improved health services for healthcare workers during times of adversity.^37^ In addition, there is a need for compassionate leadership within healthcare organisations that offers an environment of psychological safety, facilitates a sense of belonging, supports teams to prepare for and debrief from difficult events, and is attuned to the challenges that different groups of healthcare workers may face, such as cultures of presenteeism and differentials in caring responsibilities.^13,38^

### Strengths and limitations

The three main strengths of this study are its size, its breadth and the depth of participant responses. The Frontline Healthcare Workers Study is the largest survey in Australia, and one of the largest surveys in the world, to examine the experiences of healthcare workers during the COVID-19 pandemic. The survey spanned healthcare workers in all roles, from intensive care specialists to disability support workers, and settings from large hospitals to residential aged care. Finally, the free-text questions provided participants with an opportunity to write anonymously, and in detail, about their thoughts, feelings and experiences during the pandemic. Our qualitative analysis of their free-text responses offers rich insight into the experiences of this hard-to-reach group of healthcare workers.

The study has some limitations. First, data were collected at one time point, rather than longitudinally, limiting the ability to disentangle causal relationships between participants’ pandemic experiences and the onset of thoughts of suicide or self-harm. Second, the voluntary online format widened the reach of our survey, but also means that the response rate cannot be estimated. Third, some healthcare workers with frequent thoughts of suicide or self-harm may have been less likely to engage in the survey because of the severity of their mental health symptoms. Finally, we recognise the limitations of free-text question analysis.^39^ Ideally, in-depth interviews should be conducted with those who reported frequent suicidal ideation, to gain further insight into their thoughts and experiences, but there are significant limitations and cost barriers to undertaking such a study.^40^

In conclusion, the challenges of living and working during the pandemic are profound, and multipronged strategies are required to protect the well-being of healthcare workers. Our findings highlight the need for better recognition of predisposing, precipitating, perpetuating and protective factors for thoughts of suicide and self-harm among healthcare workers. These include recognising the vulnerability of healthcare workers who have a history of mental illness, who are living alone or are separated from loved ones, or who have significant stressors in their personal lives. Organisations should ensure that healthcare workers have access to adequate leave and that those who take leave are covered, rather than shifting the burden to colleagues. Ongoing efforts are needed to de-stigmatise help-seeking for mental health conditions, and to address barriers to accessing professional supports, including cost, time and confidentiality of supports. Finally, healthcare workers wanted to feel a sense of connectedness and belonging, to be able to share their psychological distress without feeling judged or afraid of adverse consequences, and to have access to skilled professional care and peer support.

These recommendations are of pressing importance for healthcare organisations whose workers continue to carry the burden of the pandemic. However, they may also be relevant for other industries seeking to protect the psychological well-being of staff, and for those working to strengthen the foundations of our healthcare system in preparation for future crisis events.

## Data availability

The authors maintain accurate records of the data associated with the manuscript. As the data-set consists of potentially identifiable free-text responses from individual healthcare workers, we are unable to provide any additional access to the data beyond the de-identified quotes included in the manuscript and tables.
